# Awareness and Attitude towards Breastfeeding among Two Generations of Indian Women: A Comparative Study

**DOI:** 10.1371/journal.pone.0126575

**Published:** 2015-05-19

**Authors:** Deeksha Pandey, Parnita Sardana, Aashish Saxena, Luvdeep Dogra, Ambika Coondoo, Asha Kamath

**Affiliations:** 1 Department of Obstetrics & Gynecology, KMC Manipal, Manipal University, Manipal, Karnataka, India; 2 MBBS Student, KMC Manipal, Manipal University, Manipal, Karnataka, India; 3 Department of Paediatrics, KMC Manipal, Manipal University, Manipal, Karnataka, India; 4 Department of Community Medicine, KMC Manipal, Manipal University, Manipal, Karnataka, India; Centre Hospitalier Universitaire Vaudois, FRANCE

## Abstract

**Introduction:**

Present study was aimed to analyze the impact of education, employment and financial independence in breastfeeding practices among Indian women.

**Methodology:**

Present explorative questionnaire based survey included 256 women (128 pairs) in the final analysis. A pair means – a) pregnant lady (in her third trimester) representing younger generation and b) her mother/mother in law representing the elder generation.

**Results:**

We found that the overall awareness regarding ‘breast milk’ being the best food for baby was excellent (overall 97.3%; younger generation: 96.9%; elder generation: 97.7%). Overall knowledge regarding the correct technique (28.9% younger generation and 21.9% elder generation) and frequency of breastfeeding (20.3% of younger generation and 34.4% of elder generation) was very poor. Less than 60% (younger generation: 57.8%; elder generation: 58.6%) were aware that the only major contraindication for breastfeeding is a mother infected with human immunodeficiency virus (HIV). On comparing responses obtained from the two generations of women, difference was not statistically significant among most of the issues related to breastfeeding. With regards to the attitude, despite better awareness, only 94.5% women in younger generation and 89.1% women in elder generation were planning to give mother’s milk as the first feed to the newborn. Similarly, less than 75% of women were ready to breast-feed the newborn immediately after birth. This was contradictory to the fact that 86% of pregnant women were aware that the baby should be breast-fed within an hour of birth.

**Conclusion:**

Awareness with regards to breastfeeding issues had not changed significantly with the educational progress of Indian women. Despite the good level of awareness in the society regarding breastfeeding, attitude to practice the same is lacking.

## Introduction

Numerous benefits of breastfeeding are well known since ages. Despite strong evidence in support of exclusive breastfeeding for the first six months of life, its prevalence has remained low worldwide. In India, according to National Family Health Survey (NFHS 3) report, only 46% under six months are exclusively breastfed and only 55% are breastfed on day one [1].

Mobility in terms of increasing awareness and educational qualifications among successive generations showcases the progress of any society. Improving women’s educational level has clear impacts on the health and economic future of the entire community. Due to the historical discrimination against women, compounded by prolonged poverty, the development of women’s education in India has been a rocky journey. In India after independence in 1947, the University Education Commission was created to recommend suggestions to improve the quality of education. Unfortunately, their report spoke against educating women. It stated that educating a woman is entirely irrelevant to the life she has to lead [2]. The literacy rate among Indian women in the post-independence era was between 2–6%. It improved to 15.3% in 1961 to 28.5% in 1981. Times are changing now. In 2001 literacy for women had exceeded 50% [3] and recent population census of India (2011) revealed it to be 65.5%.

However, the flip side of this coin is that the education has made more and more women economically independent and busy professionals. In modern India nuclear families are becoming the rule rather than an exception. We hypothesize that missing traditional family values and peer support for women might have a negative impact on the preservation of valuable breastfeeding practices in our population.

Thus, in this study we aimed to analyze the impact of increasing education, employment and financial independence in breastfeeding practices as compared to traditional family values. The awareness, attitude and practices related to breastfeeding among two generations of Indian women, was compared.

## Materials and Methods

Present explorative questionnaire based survey followed the ethical guidelines of the Institutional Review Board (Kasturba Hospital Ethics Committee) and attained its approval on 09 October 2012 (IEC 382/2012).

Primigravid women in their third trimester were enrolled in the study, only if they were accompanied by their mother/mother in law. The only criterion for exclusion was unwillingness of a lady or her mother/mother in law to participate in the study. Women (only one pair at a time) were introduced to the study by one of the investigators and were encouraged to participate in the study. A pair meant—a) pregnant woman (in her third trimester) representing younger generation and b) her mother/mother in law representing the elder generation. Women were allowed to ask questions regarding their participation, and were provided with a written summary of information about the study. The personal right to withdraw from the survey at any moment was ensured. Written consent for participation was obtained which was collected separately after it had been signed by the participant in order to avoid personal identification. Thus, anonymity and confidentiality of the participants was guaranteed.

The study was conducted in two steps. Step 1) Women were asked to answer the questionnaire having 21 items (15 related to awareness and six related to practices regarding breastfeeding). Step 2) Answers were provided by the investigator in an effort to educate the participants to make them aware of the benefits and recommended practices related to breastfeeding.

Later on, responses to the questionnaire were collated by the investigators and were analyzed statistically.

### Survey instrument

Questionnaire (S1 Text)—A questionnaire having 21 questions was prepared keeping in mind the awareness required to be disseminated to the women in relation to breastfeeding advantages and recommendations. The questionnaire contains 15 items related to awareness and six items related to practices regarding breast-feeding.

The questionnaire was developed in English. For construction and content validity, it was reviewed by five independent experts (two from Obstetrics & Gynecology, one from Pediatrics, one from Nursing, and one from Community Medicine). There was 80% agreement on the 19 questions and their wordings. In two items related to the awareness, suggested changes were made in the wordings of answer options. This English questionnaire was then translated into Kannada, which is the local language of our target population. Two experts in language then validated it for language appropriateness. The questionnaire was tested for face validity in a pilot study, on ten antenatal women to ascertain if the questions were acceptable and the wording was well understood by the respondents. ***(Kindly refer to the supplementary file: S1 Text).

#### Sample size calculation

Assuming a prevalence of 50% awareness about breastfeeding issues in the elder generation and the younger generation to have at least 15% more awareness, for a power of 80% with 95% confidence level a minimum of 99 subjects are required in each group. Accounting for a 20% non-response rate at least 124 subjects in each group need to be studied.

#### Statistical Analysis

Data was initially entered in the excel sheet (S1 Table: excel sheet with original data). This data was then analyzed using SPSS statistical software version 16. The responses of the participants to questions were analyzed according to the stratification. McNemar’s test was used to assess the significance of the responses and a P value < 0.05 was considered statistically significant.

## Results

A total of 300 women (150 pairs) were recruited for the study. However, in 12 pairs one of the women (either the pregnant woman or her mother/mother in law) did not consent to participate in the study. In 10 pairs either one or both the questionnaires were incomplete, so were excluded. As a result, 256 women (128 pairs) were included, in the final analysis.

### Demographics

Mean age of pregnant women (younger generation) in our study population was 27.4 ± 4.2 years, and their mother/mother in law (elder generation) was 52.2 ± 6.9 years. Among the elder generation 41 (32.0%) were illiterate, whereas only 2 (1.6%) were illiterate among pregnant women. Most of the pregnant women were either graduate (39.8%) or had completed their secondary education (44.5%). Among the elder generation however only one was a graduate, and most of them (46.1%) had only completed their primary education. Around 84% women of the elder generation were housewives as compared to 71% of the younger generation. (Table 1)

**Table 1:** Demographic characteristics of the population studied.

Characteristics	Mothers (n = 128)	Grand Mothers (n = 128)	p- value
**Age in years (mean** ±**SD)**	**27.4 ± 4.2**	**52.2 ± 4.2**	
**Education**			
Illiterate	02 (1.6%)	41 (32.0%)	<0.0001
Primary	18 (14.1%)	59 (46.1%)	
Secondary	57 (44.5%)	27 (21.1%)	
Graduation	51 (39.8%)	01 (0.8%)	
**Profession**			
Working (semi-skilled/skilled)	37 (28.9%)	20 (15.6%)	0.011

### Awareness related to important breastfeeding issues

We found that the overall awareness regarding ‘breast milk’ being the best food for baby was excellent (overall 97.3%; younger generation: 96.9%; elder generation: 97.7%). More than 85% women (younger generation: 89.8%; elder generation: 87.5%) agreed that for a newborn breast milk should not be supplemented with anything. Around 75% women (younger generation: 78.1%; elder generation: 73.4%) believed that colostrum should be fed to the infant. Regarding the time of initiating breastfeeding 86% of women in the younger generation as compared to 79% of women in the elder generation choose the right option of starting breastfeeding within an hour of baby’s birth. Around 80% of women in the younger generation and 73.4% of women in the elder generation believed that frequent nursing is the key factor to increase breast milk. More than 70% of women (younger generation: 76.6%; elder generation: 70.3%) were aware that supplementary feeds should be started at around six months of age. Around 60% of women in younger generation and 56% of women in elder generation agreed that breastfeeding should be completely stopped once the baby crosses two years. (Table 2)

**Table 2:** Comparison of awareness regarding initiation, cessation, frequency and technique of various aspects related to breast feeding among two generations.

Item no. (original questionnaire)	Awareness regarding various aspects of breast feeding	Correct response	Mothers (% of correct response) N = 128	Grand Mothers (% of correct response) N = 128	Statistical s significance (p value)
1.	Best food for newborn	Mothers milk	124 (96.9%)	125 (97.7%)	1.000
2.	Supplementation with breast milk (initial days)	Nothing	115 (89.8%)	112 (87.5%)	0.694
3.	What should be done with Colostrum	To be fed to the baby	100 (78.1%)	94 (73.4%)	0.486
4.	Breast feeding should be started	Within one hour	110 (85.9%)	101 (78.9%)	0.189
5.	Quantity of breast milk is increased with	Frequent nursing	102 (79.7%)	94 (73.4%)	0.302
7.	Frequency of breast feeding	On demand	26 (20.3%)	44 (34.4%)	**0.017**
8.	Technique of breast feeding	Full areola in baby’s mouth	37 (28.9%)	28 (21.9%)	0.251
9.	Start of supplementary feeds	6 Months	98 (76.6%)	90 (70.3%)	0.322
15.	Complete cessation of breast feeding	2 years	78 (60.9%)	72 (56.2%)	0.526

In our study, the overall knowledge regarding the correct technique and frequency of breastfeeding was very poor. Only 20.3% of women in younger generation and 34.4% of women in elder generation knew that breastfeeding is best practiced on demand basis. Only 28.9% women in younger generation and 21.9% women in elder generation knew the correct technique of breastfeeding. The difference of awareness with regards to frequency and technique of breastfeeding was statistically significant among the two generations (P value <0.05). (Table 2).

Less than 60% (women in younger generation: 57.8%; women in elder generation: 58.6%) were aware that the only major contraindication for breastfeeding is if mother is infected with human immunodeficiency virus (HIV). Others believed breastfeeding should be withheld if the baby is born by cesarean, mother is febrile, or baby is small/sick. More than two third women in the study population (younger generation: 71.7%; elder generation: 69.5%) were aware that there are no side effects of frequent breastfeeding (like child obesity, postpartum depression and deterioration in milk quality). (Table 2)

Only a few participants knew about the gross composition of mother’s milk. While 60–70% of women were aware of the advantages of breastfeeding for the baby, only 50.8% women in younger generation and 43.1% women in elder generation knew that feeding the newborn can prevent breast cancer among mothers in later life. (Table 3)

**Table 3:** Comparison of awareness regarding contraindications, advantages and disadvantages of breast feeding to the child and the mother.

Item no. (original questionnaire)	Awareness regarding various aspects of breast feeding	Correct response	Mothers (% of correct response) N = 128	Grand Mothers (% of correct response) N = 128	Statistical s significance (p value)
6.	Breast milk is contra indicated in	Mother with HIV/AIDS	74 (57.8%)	75 (58.6%)	1.000
10.	Side effects of breast feeding	None	91 (71.1%)	89 (69.5%)	0.891
11	Components of breast milk	Galactose, Protein, Fat, Immunity particles	27 (21.1%)	21 (16.4%)	0.424
12.	Advantages of breast milk for baby	Balanced nutrition, easily digestable, reduces infection	78 (60.9%)	81 (63.3%)	0.797
13.	Preventive benefits of breastfeeding for baby	Diarrheal diseases, Ear infection, Respiratory infection	88 (68.8%)	81 (63.3%)	0.429
14.	Advantages of breastfeeding to mother	Prevention against breast cancer	65 (50.8%)	55 (43.0%)	0.260

### Breastfeeding practices

**Fig 1:**
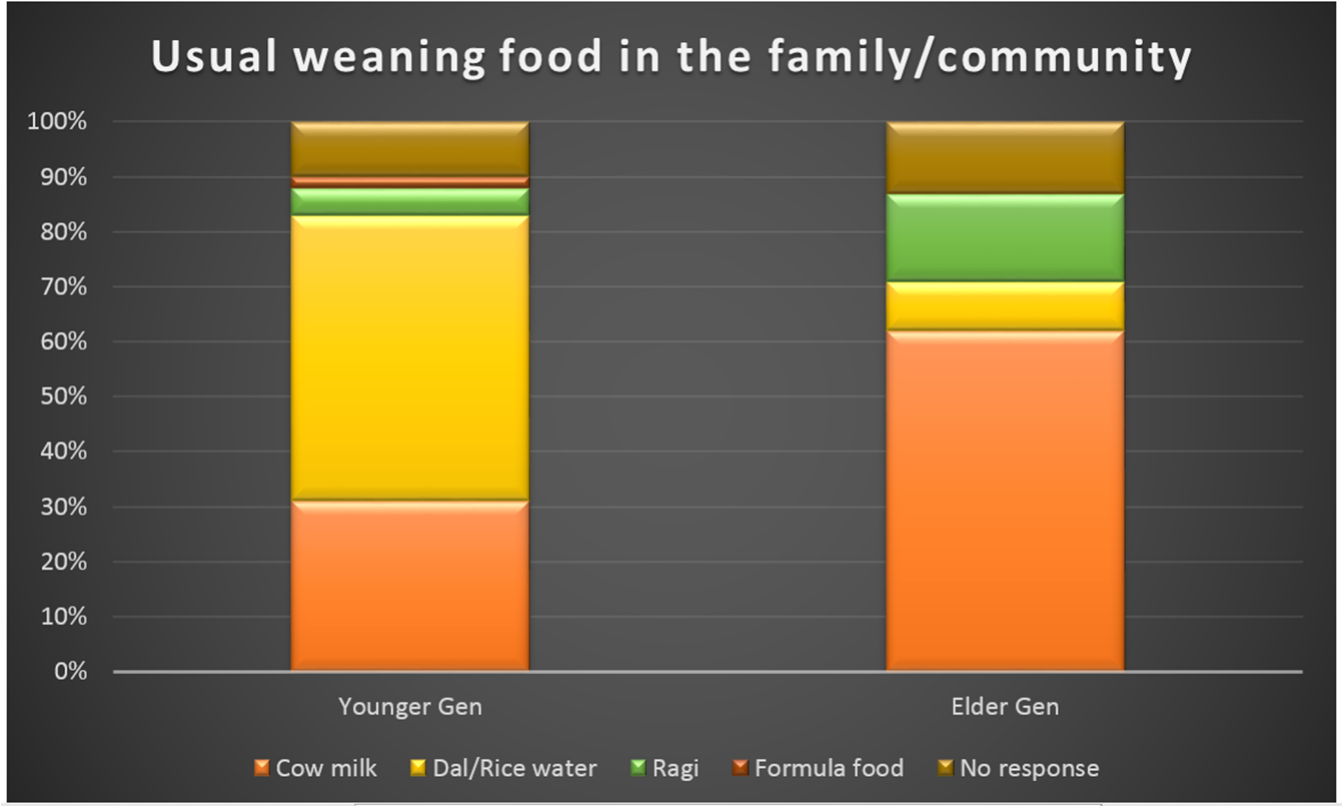
Responses obtained from the two groups when asked about the usual weaning food in their family/community. (*Dal: Pulses, *Ragi: Finger Millet: these two are commonly used in Indian population).

Though around 98% of women in our study group were aware that breast milk is the best first food for the newborn; fewer were ready to practice it. Only 94.5% women in younger generation and 89.1% women in elder generation were planning to give mother’s milk as the first feed to the newborn. More than half (52%) women in younger generation told that the usual weaning food in their family/community was commercially available formula food. Around 62% of women in elder generation told that the usual weaning food was cow’s milk, in their household. (Fig 1)

One-third (34.5%) of women had never discussed with anyone regarding the feeding practices for the ‘baby to be born’. About 40% of pregnant women had discussed this issue with some of their family member. Surprisingly only around 7% of women accepted that their obstetrician had already discussed this issue with them, during antenatal visits.

**Fig 2:**
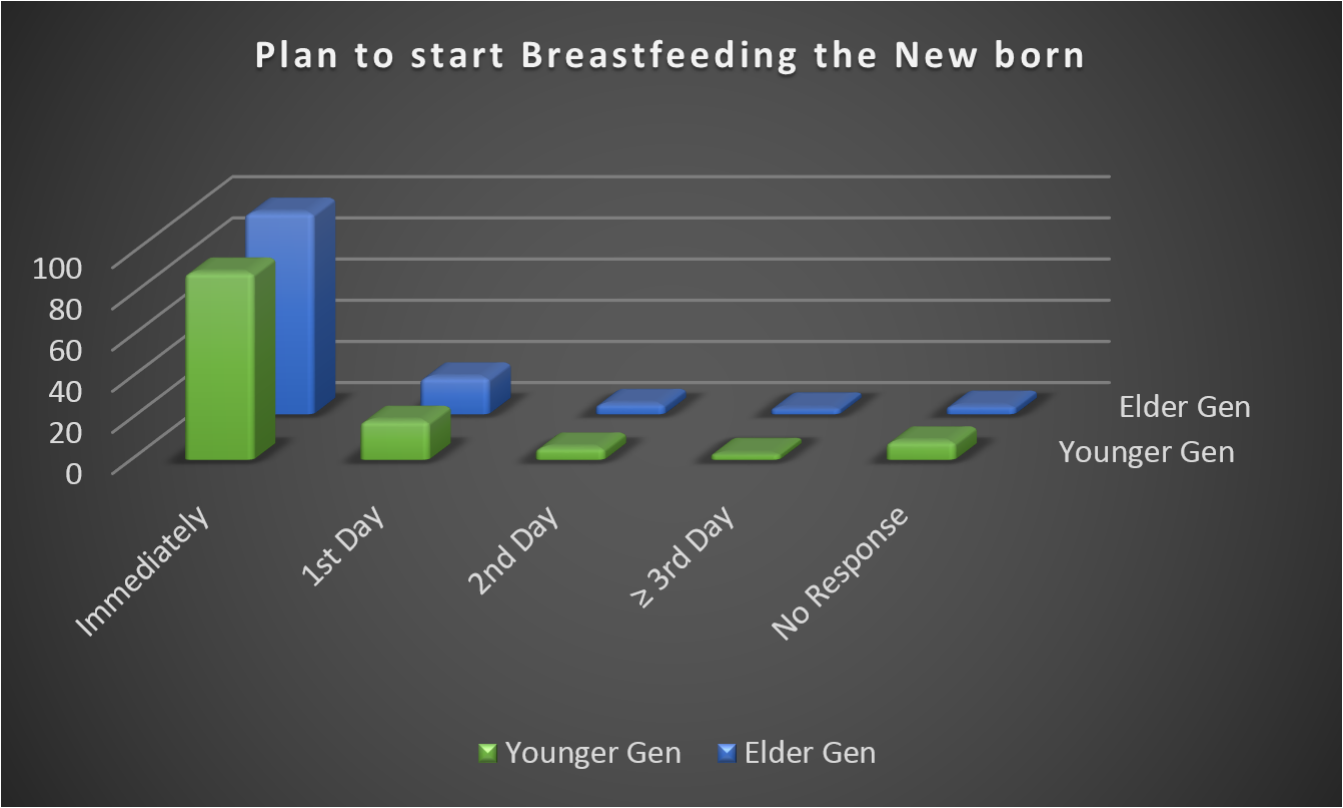
Responses obtained from the two groups when asked about the plan of time of initiating breast feeding.

On an average, less than 75% of women were ready to breast-feed the newborn immediately after birth (Fig 2). This was contradictory to the fact that 86% of women in younger generation and 79% of women in elder generation were aware that the baby should be breast-fed within an hour of birth.

**Fig 3:**
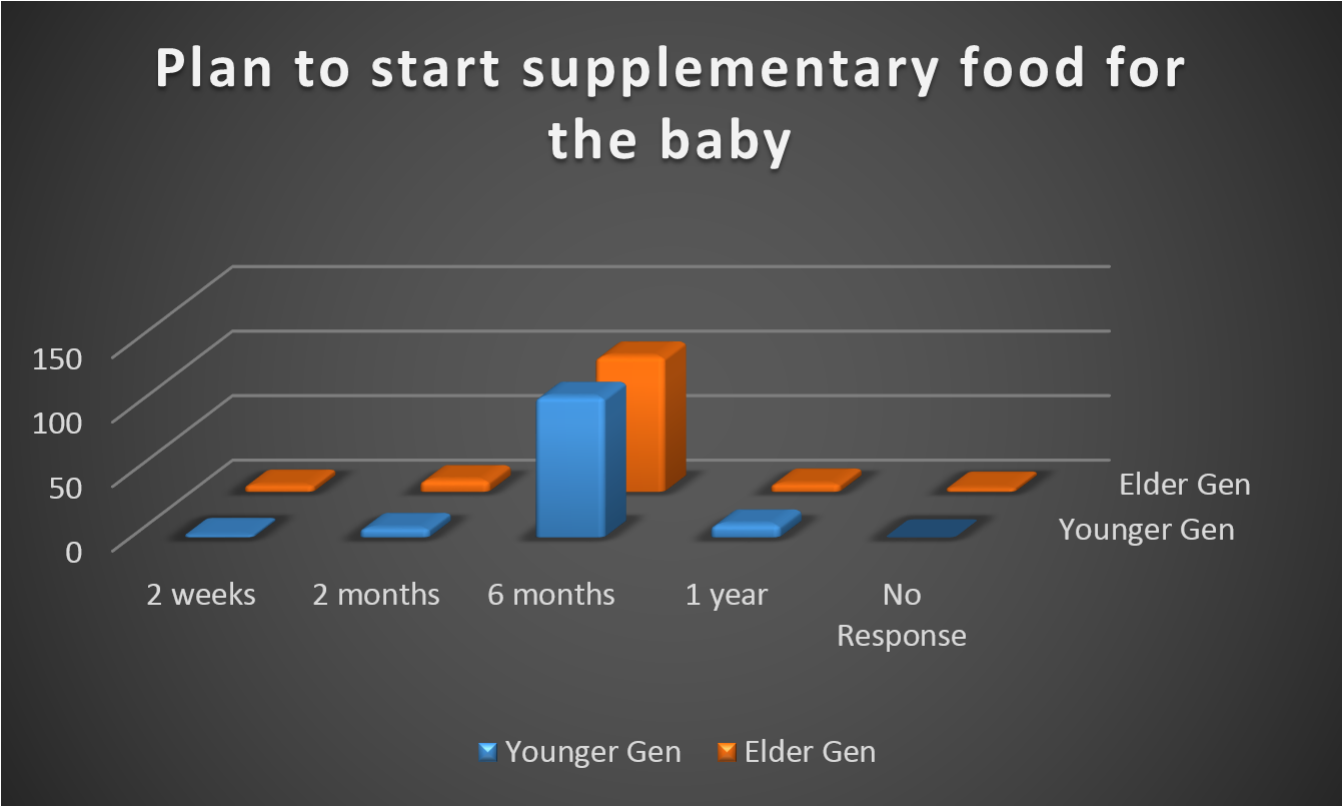
Responses obtained from the two groups when asked about the plan to start weaning (supplementary food) for the baby.

As depicted in the figure, more than 80% of women (85.4% younger generation, 81.8% elder generation) were planning to start weaning the baby by six months of age (Fig 3).

## Discussion

As hypothesized we didn’t find statistically significant difference in awareness regarding most of the breastfeeding issues among the two generations of Indian women. The study also revealed that despite the good level of awareness in the society regarding breastfeeding, attitude to practice the same is lacking. Similar mismatch was found in another study from India, where 100 postnatal mothers with a mean age of 25.18 years were enquired regarding breastfeeding related issues. In this cohort while 92% of women knew that breastfeeding should be initiated within one hour, only 36% had done so [4].

A study from Ghana with a large sample size of 10,947 infants concluded that the promotion of early initiation of breastfeeding has the potential to make a major contribution. Up to 16% of neonatal deaths can be saved, if all infants are breast-fed from day one. Up to 22% of neonatal deaths can be prevented if breastfeeding is started within the first hour of birth [5]. Sadly, optimizing breastfeeding practices, has not been understood as one of the most effective interventions to reduce infant and young child mortality, morbidity and malnutrition [6].

In our study 75% women believed that colostrum should be fed to the infant while other studies from India has a wide range of awareness (56–90%) in this regard [4,7,8]. Preparation of mothers during the antenatal period is fundamental to the success of exclusive breastfeeding. In our study, we found that only around 35% of women had a discussion regarding breastfeeding issues during pregnancy, and unfortunately only 7% of women were informed regarding this by their obstetrician. Likewise in another Indian study more than half (52%) of the women had not received any advice regarding breastfeeding during their antenatal period. Only 17% received information on this from the health care workers [4]. It is understood that in a densely populated country like ours we definitely have a great burden on us while delivery the obstetric care. However, the fact cannot be ignored that preparing the lady for breastfeeding that has been recognized as one of the best intervention to reduce infant mortality and morbidity, is also our responsibility. Obstetrician should become more proactive in this regards while delivering the other routine antenatal services. Experts have even proposed to target young women early during school years to increase their awareness of breastfeeding [9]. In our population, more than 70% of women knew about exclusive breastfeeding for six months. In a Nigerian study despite the knowledge regarding breastfeeding was abysmally low all the children were breast-fed because of the tradition [10].

On comparing the awareness of two generations of women, we realized that the difference was not statistically significant among most of the issues related to breastfeeding (except for frequency of breast feeding). Though the difference was statistically not significant, the younger generation seemed to be more aware of the benefit of colostrum, and initiating of breastfeeding within an hour of birth. There was a trend towards more awareness related to indirect benefits of breastfeeding to the mother, and the correct technique of breastfeeding in the elder generation.

In the step 2) of the study after the questionnaire was filled, along with reinforcement of already known facts we also tried to address optimal frequency and technique of breastfeeding to the pregnant women. The importance of correct positioning, and achieving latch-on was also emphasized.

We enrolled in the study only those women who were accompanied by their mother/mother in law. The reason for this kind of study was that most of the Indian women in our setup prefer to come with their mother/mother in law for their antenatal check-up. Despite the level of awareness the practices are usually based on the tradition, which is also evident in the present study. However this might be one of the caveats in our study as women who are more educated and know more about appropriate breastfeeding techniques are also more likely to go to their obstetrics appointments without needing a chaperone.

In a study among Singaporean mothers giving birth from 2000 to 2008, breastfeeding initiation and duration increased over time and were independently associated with higher maternal education [11] In a Malaysian study however it was reported that women who were working and belonged to higher socio-economic status were less likely to exclusively breastfeed their infants [12] Survey done on the Ethiopian immigrant mothers in Israel also concluded that conventional family values need to be reinforced to preserve the traditional breastfeeding practices in the community [13]. In our knowledge, this is the first study where we have compared two generation of women with regards to their awareness and attitude towards breastfeeding. In our cohort women of younger generation were more educated and more financially independent professionals as compared to the elder generation. Still the level of awareness was not statistically different. However in both the groups the attitude towards good breastfeeding practices was low.

## Conclusion

It can be concluded from the present study that attitudes and awareness with regards to breastfeeding issues have not changed significantly with the educational progress and economic independence among the two generations of Indian women. Despite the good level of awareness in the society regarding breastfeeding, attitude to practice the same, is lacking. Obstetrician should become more proactive in this regards while delivering the other routine antenatal services.

## Supporting Information

S1 Text Questionnaire which was used as the survey instrument in the study. (DOC)

S1 Table Excel sheet with the original data. (XLSX)
