# Supplementary material for: Awareness and Attitude towards Breastfeeding among Two Generations of Indian Women: A Comparative Study
Source: PLoS One. 2015 May 19;10(5):e0126575. doi: 10.1371/journal.pone.0126575 (PMC4437785; doi:10.1371/journal.pone.0126575)
Supplement: S1 Text — (DOC) [file pone.0126575.s001.doc]

**Awareness and attitude towards breast feeding among two generations of Indian women: A comparative study**

**Study no (official use only)**

**Please answer the questions by ticking the box after your chosen response**

**Age:**

**Relation to the ‘would be born’: Child: Grand Child:**

**Educational Status:**

**Socio Economic Status:**

**Occupation:**

**Religion:**

***Parity of the pregnant lady**

***EDD: POG:**

**Remarks**

1. **Best food for a new born baby is**

**Cow milk Honey Mother’s milk Water**

1. **In the initial few days after birth breast milk should be supplemented with**

**Cow milk Hot Water Honey Nothing**

1. **Colostrum (the yellowish discharge from milk during first days after child birth) should be**

**Discarded Fed to the baby**

**Mixed with cow milk and given to the baby Mixed with honey and given to the baby**

1. **When should a mother start breast feeding her new born baby**

**Within 1h After 6h Next day After 2 days**

1. **What increases the quantity of breast milk?**

**Frequent nursing Milk intake by mother**

**Dry fruits Medication**

1. **Breast milk should not be given to the baby in following situation**

**After cesarean delivery If mother has HIV/AIDS**

**If mother has fever If baby is small /sick**

1. **Breast feeding should be done**

**every hour every 4h 4 times a day On demand**

1. **When breast feeding the baby**

**Only nipple should be inside baby’s mouth**

**Half of the areola should be inside baby’s mouth**

**Full areola should be inside baby’s mouth**

**Full breast should be inside baby’s mouth**

1. **When should the baby be started on supplementary feeds?**

**2 weeks 2 months 6 months 1 year**

1. **Frequent breast feeding can cause**

**Low quality milk Postpartum depression**

**Child obesity None of the above**

1. **Which of the following is not a component of breast milk?**

**Fat Hormones Immunity particles Protein**

1. **Breast milk has the following advantages for the baby**

**Provides balanced nutrition**

**Reduces immunity and infection risks**

**Is easily digested and absorbed**

**All of the above**

1. **Breast feeding prevents the baby from**

**Diarrheal disease Ear infection**

**Respiratory infection All of these**

1. **Breast feeding protects the mother from**

**Breast cancer Infections Hypertension Anemia**

1. **When breast feeding should be completely stopped?**

**when the baby starts taking food adequately 6 months of age**

**1 year of age 2 years of age**

1. **What is the usual weaning food in your family/community?**

**Cow milk dal/rice water ragi formula food**

1. **Has anyone of the following discussed with you regarding the feeding practice for this ‘would be baby’?**

**Obstetrician Nurse Family member None**

1. **Would you like yourself to be educated by experts?**

**Yes No**

1. **What are you planning to give newborn as first food?**

**Cow milk Honey Mothers Milk Others please specify ____________**

1. **When are you planning to start breast feeding?**

**Immediately after birth**

**1^st^ day**

**2^nd^ day**

**More than 3 days**

1. **When are you planning to start supplementary foods for the baby**

**2 weeks**

**2 months**

**6months**

**1 year**

***Thank you for your participation***

***All information obtained in the study will be kept Confidential and used for medical research only***
